# Overview of strategies to improve the antibacterial property of dental implants

**DOI:** 10.3389/fbioe.2023.1267128

**Published:** 2023-09-27

**Authors:** Shaobo Zhai, Ye Tian, Xiaolu Shi, Yang Liu, Jiaqian You, Zheng Yang, Yuchuan Wu, Shunli Chu

**Affiliations:** Jilin Provincial Key Laboratory of Tooth Development and Bone Remodeling, Hospital of Stomatology, Jilin University, Changchun, China

**Keywords:** dental implant, antibacterial property, surface modification, coatings, surface topography

## Abstract

The increasing number of peri-implant diseases and the unsatisfactory results of conventional treatment are causing great concern to patients and medical staff. The effective removal of plaque which is one of the key causes of peri-implant disease from the surface of implants has become one of the main problems to be solved urgently in the field of peri-implant disease prevention and treatment. In recent years, with the advancement of materials science and pharmacology, a lot of research has been conducted to enhance the implant antimicrobial properties, including the addition of antimicrobial coatings on the implant surface, the adjustment of implant surface topography, and the development of new implant materials, and significant progress has been made in various aspects. Antimicrobial materials have shown promising applications in the prevention of peri-implant diseases, but meanwhile, there are some shortcomings, which leads to the lack of clinical widespread use of antimicrobial materials. This paper summarizes the research on antimicrobial materials applied to implants in recent years and presents an outlook on the future development.

## 1 Introduction

With the development of implant materials and technologies, dental implants have become the choice of more and more patients with missing teeth as they can not only better meet the aesthetic requirements without damaging the adjacent teeth, but also maintain a high success rate while maximizing the restoration the patient’s masticatory function. In recent years, the number of dental implants worldwide has increased exponentially, and the global implant market is expected to reach $13.01 billion by 2023 (Alghamdi and Jansen, 2020). However, with the popularity of dental implants, the number of those who suffer from peri-implant diseases is also increasing, causing a great deal of distress to patients and doctors. Peri-implant disease is defined as inflammatory damage that occurs in the soft and hard tissues surrounding the dental implants, including peri-implant mucositis and peri-implantitis. A recent study reported that dental implants have a 10-year survival rate of 96.4% (Howe et al., 2019). However, 54.7% of patients suffer from peri-implant mucositis and 22.1% of patients suffer from peri-implantitis during an average of 23.3 years after receiving dental implants treatment (Renvert et al., 2018). Another study showed that 47% of implant treatment failures are related to peri-implantitis (Amer and Sukumaran, 2021). So peri-implant diseases largely determine whether the implant treatment can be successful.

**FIGURE 1:**
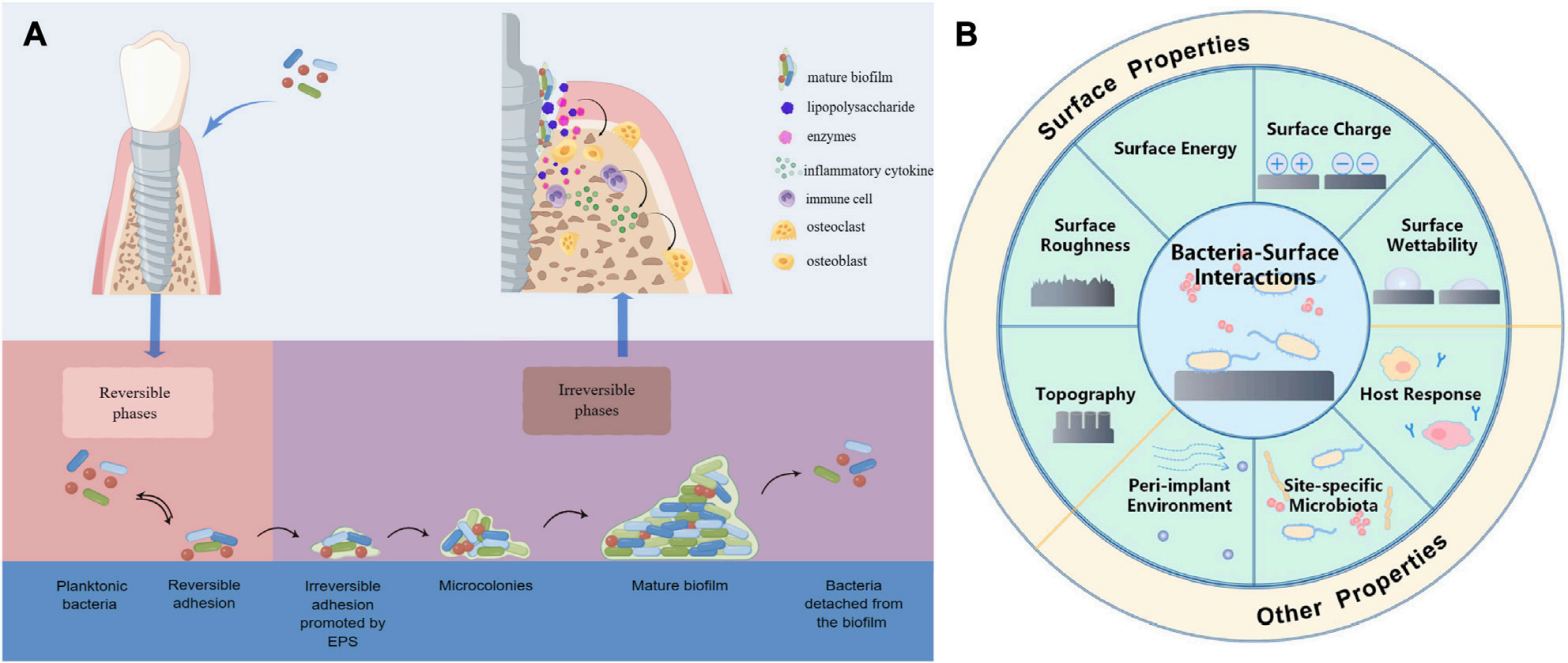
**(A)** Schematic presentation of biofilm formation process and bacteria secreting lipopolysaccharides and enzymes that act on osteoblasts and immune cells to cause them to secrete inflammatory factors that promote osteoclast formation, leading to bone resorption. **(B)** Schematic presentation of the various factors affecting bacterial adhesion. Reproduced with permission from (Ma et al., 2022). Copyright (2022) Frontiers Media.

Peri-implantitis is a pathological condition that occurs in the supporting tissues around dental implants and is characterized by peri-implant mucosal inflammation and progressive supporting bone loss, which, combined with its early onset and non-linear and accelerated pattern of progression, can eventually lead to the failure of dental implants without intervention and treatment (Fu and Wang, 2020). It is well known that plaque colonized on the implant is the initial factor of peri-implantitis and that microorganisms have a significant role in the occurrence and progression of peri-implantitis through formation of biofilm. After the implant surface is exposed to the oral environment, bacteria begin to adhere and colonize to the conditional film formed by salivary components on the surface of the implant and form a biofilm (Chen et al., 2021; Ardhani et al., 2022). The maturation of the biofilm undergoes the following phases: (1) cells reversibly attach to the surface; (2) extracellular polymeric substance (EPS) secreted by cells promote the irreversible attachment to the surface; (3) cells that attach to the surface replicate and form microcolonies of tens to hundreds of micrometers; (4) with the replication of cells and the accumulation of EPS, mature biofilms with three-dimensional structures are formed; (5) some cells detach from the biofilm and disperse into the surrounding liquid environment, absorbing on the surface and generating new biofilm (Renner and Weibel, 2011) (Figure 1A). After biofilm is formed, it is difficult for us to remove it by conventional methods because the bacteria in the biofilm have stronger resistance to the body’s defense mechanisms as well as to antimicrobial drugs compared planktonic bacteria (Del Pozo, 2018). Therefore, therapeutic approaches that intervene bacterial adhesion and biofilm formation are expected to be one of the effective ways to prevent peri-implant diseases in the future.

There are many factors that influence the interaction between bacteria and the surface, including the bacteria associated factors, the surface properties of the materials, and environmental factors (Figure 1B). Surface modification of the implant is one of the effective ways to reduce bacterial adhesion, and the surface properties of the implant are critical to the adhesion of the tissue as well as the formation of biofilm. Bacteria are able to perceive chemical signals and surface-associated mechanical signals, according to research conducted *in vivo* and *in vitro*, so different implant surface properties can have different effects on bacterial adhesion, physiology, and formation of biofilm (Renner and Weibel, 2011; Bermejo et al., 2019). For example, *Streptococcus* salivarius (S. salivarius) is specifically bound to the tongue but not to the teeth by microvilli structures on the tongue surface, whereas the binding site of *Streptococcus* mutans (S. mutans) is opposite to that of S. salivarius (Beachey, 1981). The majority of oral implant materials are presently constructed of pure titanium (Ti) and titanium alloys (Ti_6_Al_4_V), as the metal Ti have a range of desirable qualities, including good biocompatibility, corrosion resistance, and osseointegration ability (Delgado-Ruiz and Romanos, 2018). However, the conventional Ti implants that are utilized in clinical practice lack excellent antibacterial properties, and in recent years, researchers have been working to develop new implant materials with better antibacterial properties, such as Zirconia, alloys, *etc.*, or by changing the surface properties of the implant to obtain better antimicrobial properties, such as surface topography (surface roughness, contour shape, *etc.*), chemical composition, surface wettability, surface energy, surface charge, *etc.* This paper presents a comprehensive summary of the research on implant surface modification and antimicrobial materials used to enhance the antibacterial properties of implants in recent years to provide a theoretical basis for future prevention of peri-implant diseases.

## 2 Antimicrobial coatings

Researchers have made extensive research on the addition of coatings with antimicrobial properties on the surface of implants to resist bacterial adhesion, colonization, migration, and biofilm formation. The coatings commonly used to enhance the antimicrobial properties of implant surfaces include some metallic elements, antibiotics, antimicrobial peptides, chlorhexidine, synthetic polymers and so on.

### 2.1 Metal elements

Some metallic materials have excellent antimicrobial activity and are widely used for enhancing the antimicrobial properties of implant surfaces. The most commonly used metallic materials are silver, copper and zinc.

As the most potent antimicrobial metal element, silver (Ag) has broad-spectrum antibacterial properties and little toxicity to human beings (Lampé et al., 2019). It can provide anti-microbial action both in Ag^+^ form and through directly "contact killing". The antimicrobial power of Ag is mainly expressed by the form of Ag^+^. Ag^+^ is able to enter the cytoplasm of bacteria and exert its antimicrobial action by interfering with the function of a variety of proteins and enzymes and restraining the synthesis of nucleic acids and proteins, leading to bacterial death, and Ag nanomaterials also function as antimicrobials by releasing Ag^+^ (Eliaz and Metoki, 2017; Wei et al., 2020; Liu et al., 2023). Another significant mechanism by which Ag kills bacteria is direct contact killing. Ag disrupts bacterial cell walls and cytoplasm membranes through contact with bacteria, which results in leakage of cytoplasmic and essential cellular substances and ultimately leads to the death of bacteria (Bui et al., 2019). In addition, Ag nanoparticles (Ag NPs) are able to provide stimulation of soft tissue integration and osteogenesis, which makes them a desirable choice for surface modification of dental implants. A study showed that nano-Ag coating prepared on pure Ti using microwave-assisted synthesis significantly inhibited the aggregation of *S. aureus* (*Staphylococcus aureus*) on the surface (CFUs of on nano-Ag-coated Ti disks and control Ti disks are 30 ± 15.1 and 168.3 ± 32.9, respectively), *in vivo* and *In vitro*, they exhibited good antibacterial properties and no significant cytotoxic effects were observed (Odatsu et al., 2020). Li et al. prepared a time-dependent and self-adjusting antimicrobial coating by incorporating Ag nanoparticles as antibacterial agents deeply into the bottom of TiO_2_ NTs prepared on pure Ti surface through centrifuge. The Ag NPs can automatically convert to an immobile state from the free state, in the early phase, the coating released Ag ions exhibiting strong "release bactericidal" activity, and gradually changed to "contact bactericidal" activity. This special design meets the antimicrobial requirements of the implant at different times after implantation and effectively resists both planktonic and immobilized bacteria (Li B. et al., 2019). Ag has excellent antimicrobial properties, but the extensive use of silver antimicrobial agents and the increasing resistance of bacteria have led to the emergence of silver-resistant bacteria. There are several reports on bacterial antimicrobial resistance to ionic Ag, and it is generally believed that bacteria resist the antimicrobial effect by eliminating Ag(I) through efflux pumps or by reducing it to less toxic Ag (0) oxidation state. There are fewer studies on bacterial resistance to AgNPs. There is no definite conclusion on the mechanism of its resistance, and some scholars believe that its resistance originates from the production of flagellin, which can lead to the aggregation of AgNPs, thus eliminating its antibacterial effect (Stabryla et al., 2021). Some scholars believe that its resistance is not related to the aggregation of AgNPs, and its resistance mechanism may be enhanced or mediated by flagellum-based motility (Panáček et al., 2018). However, it is certain that bacteria repeatedly exposed to sub-inhibitory concentrations of AgNPs can acquire stable resistance. In addition, there are concerns about toxicity due to the accumulation of metal ions, and more research is needed to identify the optimal Ag concentration required to kill bacteria and the mechanisms by which bacteria develop resistance, in order to rationalize the use of Ag antimicrobials and reduce the development of resistance.

**FIGURE 2:**
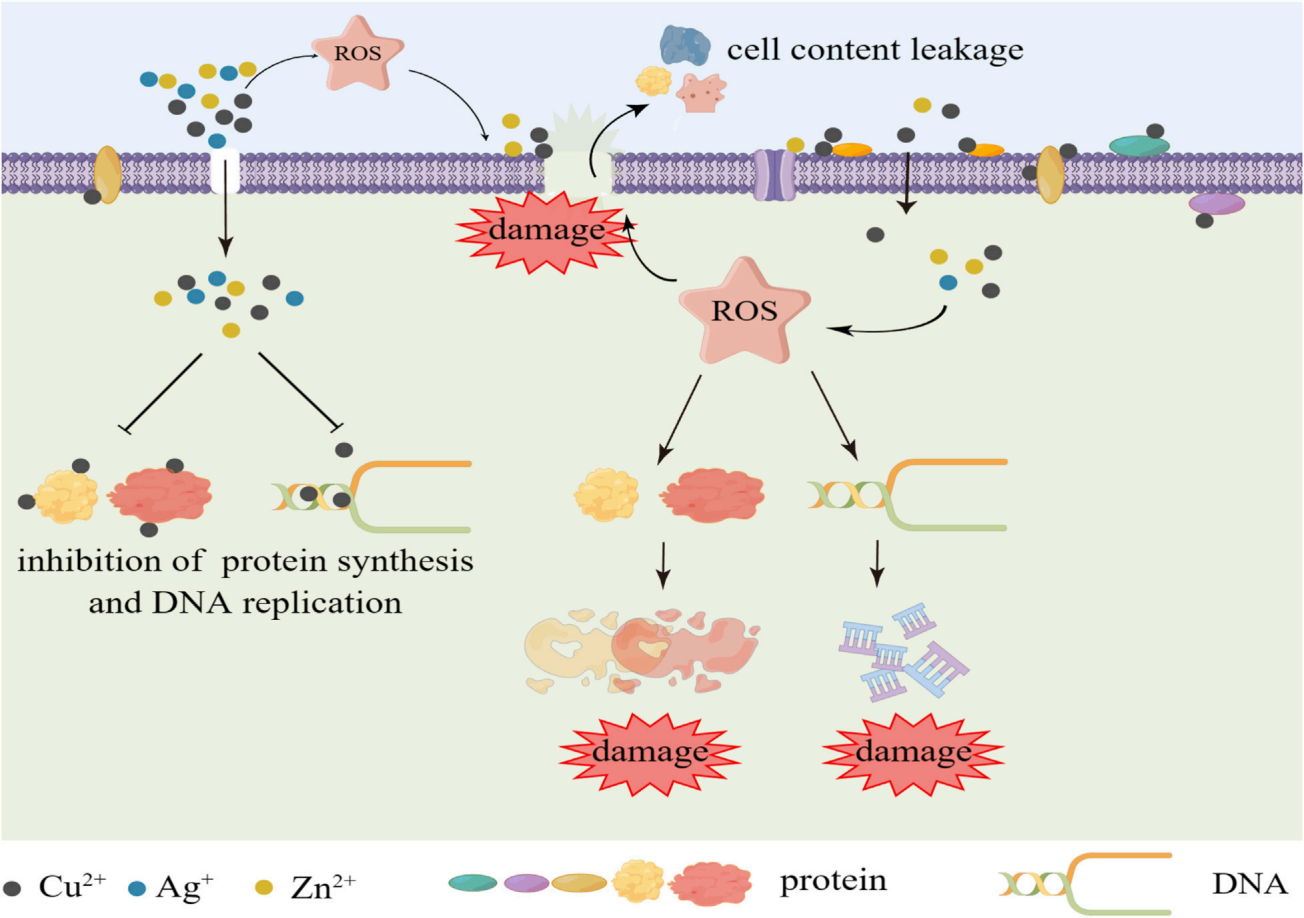
Schematic presentation of the bactericidal mechanism of Cu ion, Ag ion and Zn ion.

Copper (Cu) is an essential trace element for human body with well recognized antibacterial and antiviral properties, capable of inactivating a wide range of bacteria and viruses, such as *S. aureus*, *E. coli* (*Escherichia coli*), SARS-CoV-2, influenza, *etc.* (Li et al., 2016; Aissou et al., 2023), and Cu NPs also have osteogenic and angiogenic properties (Burghardt et al., 2015). Compared to Ag, this metal is cheaper and more readily available, providing an effective antimicrobial effect over a wider range of temperatures and humidity, and also has “contact killing” mechanism. Cu induces the production of reactive oxygen species (ROS) through Fenton-like reactions, which result in damage to lipids, proteins, DNA, and cell membranes. Cu ions can enter bacteria through membrane channels and repress the synthesis of relevant enzymes and activity of bacterial DNA and interfere with the metabolism of bacteria (Figure 2). Cu nanoparticles (CuNPs) in Ti implants could release Cu ions to suppress the activity of bacteria (Wang et al., 2019; Aissou et al., 2023). Jannesari et al. have demonstrated that Cu ions and its superoxide can exert excellent antibacterial properties by suppressing the respiration of bacteria and causing DNA breakdown (Jannesari et al., 2020). Moreover, Cu has also been found to be the most effective metal to prevent the biofilm formation of *E. coli*, *S. aureus*, *P. aeruginosa* (*Pseudomonas aeruginosa*), etc (Gugala et al., 2017). Xia et al. reported the modification of Ti implants by co-implantation of C/Cu nanoparticles using plasma immersion ion implantation and deposition (PIIID) technique. While showing excellent corrosion resistance and mechanical properties, the antimicrobial properties of the modified implants were significantly enhanced, with the C/Cu-Ti surfaces achieving 90% and 100% inhibition against *S. aureus* and *E. coli*, respectively (Xia et al., 2020). van Hengel et al. used plasma electrolytic oxidation (PEO) to incorporate different proportions of Cu and/or Ag NPs into Ti dioxide layer prepared on Ti-6Al-4V surface. The modified implants exhibited strong antimicrobial activity, and in an *ex vivo* model using murine femora, all bacteria were eradicated by the functionalized implants with 25% Cu and 75% Ag NPs, and the implants containing just Cu NPs enhanced the metabolic activity of pre-osteoblastic MC3T3-E1 cells (van Hengel et al., 2020). Peri-implantitis induces ROS production, and the accumulation of excess ROS disrupts redox microenvironmental balance, inducing biofilm formation and immune disorders. Cu^2+^ induces ROS production while killing bacteria, further exacerbating redox imbalance and immune disorders, and ultimately impeding infection clearance and tissue repair, which is a cause for our concern. With this consideration, Xu et al. designed a luteolin (LUT)-loaded copper (Cu^2+^)-doped hollow mesoporous organosilica nanoparticle system (Lut@Cu-HN), which utilized LUT to scavenge excess ROS to prevent Cu^2+^ from exacerbating the redox imbalance and reducing the immunotoxicity of Cu^2+^, and showed excellent antimicrobial and immunomodulatory activities in in vitro and *in vivo* experiments (Xu et al., 2023). Therefore, while focusing on the bactericidal effect, we should also pay more attention to the balance of the microenvironment around the implant and the regulation of immune homeostasis.

Zinc (Zn), as an essential trace element, also has desirable antimicrobial properties and has been widely applied in oral treatment as an effective antibacterial agent. Although its antibacterial activity is weaker than that of Ag and Cu, it has many advantages as an antibacterial agent, such as high safety, low side effects, and long-lasting antibacterial effect. Zn ions can interact with cell membranes, disrupt bacterial cell membranes and intracellular proteins, inhibit the activity of related enzymes, and produce multiple antibacterial effects on bacteria (Li et al., 2013; Almoudi et al., 2018). The antimicrobial mechanisms of Zn oxide include photocatalytic antibacterial mechanism and contact sterilization mechanisms causing surface damage to bacteria by interacting with the bacterial membrane, as well as inducing the production of ROS, which ultimately results in death of bacteria (Xie et al., 2011; Liu W. et al., 2014; Memarzadeh et al., 2015). In addition, Zn can affect the function of osteoclasts and osteoblasts, reducing bone resorption and promoting bone formation (He et al., 2023). Zhang et al. prepared a macroporous Ti dioxide coating by micro-arc oxidation (MAO), and introduced Zn into the modified Ti surface by hydrothermal treatment to prepare a novel TiO_2_/ZnO micro/nanostructured coating, followed by a simple heat treatment to improve its cytocompatibility, and the Zn-doped TiO_2_ coating exhibited a powerful inhibitory ability against *S. aureus*, which could reach an antibacterial rate of 96.1% ± 3.3% (Zhang et al., 2018). However, efficient antimicrobial activity is usually accompanied by cytotoxicity, and enriched Zn^2+^ on the surface can also be detrimental to angiogenic and osteogenic on the implant surface. Therefore, a balance between antibiotic activity accompanied by a certain degree of cytotoxicity and angiogenic and osteogenic needs to be achieved. Wang et al. applied Ti plasma immersion ion implantation (Ti-PIII) to modify carbon fiber reinforced polyetheretherketone (CFRPEEK), then hybrid polydopamine (PDA) @ZnO NPs was modified onto CFRPEEK via PDA-assisting covalent immobilization, and pro-angiogenic Endothelin-1 (EDN1) was further doped onto the surface to balance the adverse effects of antimicrobials on osteointegration. *In vitro* and *in vivo* experiments showed that the modified surface exhibited excellent bactericidal ability against *S. aureus* and *E. coli*, while effectively promoting angiogenesis and osteogenesi (Wang X. et al., 2023).

In addition to Ag, Cu, and Zn, metallic elements such as cerium, gallium, zirconium, and bismuth have also been explored for surface modification of implants. Some non-metallic materials such as fluorine, silicon, graphene, and hydroxyapatite have also been explored as antimicrobial materials or carriers of antimicrobial materials to improve the antimicrobial properties of implants. Koopaie et al. demonstrated that the addition of zirconium, silicon, and fluorine to the surface of SLA-treated γ-TiAl significantly improved its antimicrobial activity against Actinobacillus actinomycetemcomitans (AA), Eikenella corrodens (EC) (Koopaie et al., 2020). Jang et al. also demonstrated that graphene oxide coating deposited on zirconium oxide surface significantly inhibited the adhesion and biofilm formation of S. mutans (Jang et al., 2021). While each antibacterial agent or element presents its distinctive antibacterial properties, it also has its strengths and weaknesses. For example, Ag NPs may cause cytotoxicity by producing ROS, releasing free Ag + ions, transporting across the blood-brain barrier, and inflammation (Noronha et al., 2017), the aggregation of Zn could induce mammalian cells cytotoxicity, and ZnO NPs may lead to damage of DNA and apoptosis or necrosis of cells (Kononenko et al., 2017). The toxicity can be reduced by decreasing the dose of the corresponding metal ion, and in addition, the use of multiple antibacterial agents or elements in combination has been explored in order to achieve the desirable antibacterial effect, as well as excellent osteogenic properties and biocompatibility. Li et al. simultaneously implanted different ratios of Zn and Ag ions into titanium nitride (TiN) on Ti alloy surface by plasma immersion ion implantation (PIII) system and obtained good antibacterial ability while the inhibitory effect of adhesion and proliferation of fibroblast-like cells by Ag was compensated as the ratio of Zn increased (Li L. et al., 2019). Therefore, the use of a variety of antimicrobial agents to exert synergistic antimicrobial effects, improve the biocompatibility of surface, and develop better drug controlled release system may be one of the important development directions in the future.

### 2.2 Polymers

Polymeric layers have attracted much attention for antibacterial properties and the enhancement of bioactivity and effective control of potent drugs release, and different polymers have been used by researchers as surface coatings for implants to improve their antibacterial properties. Some polymers can produce coatings on the surface of implants that prevent bacterial adhesion, called antifouling polymers. These polymer coatings, which reduce the adhesion of proteins and bacteria rather than directly killing the bacteria in contact, include mainly hydrophilic and amphoteric polymers. These two polymers can provide antifouling effects by creating a hydrated layer on the surface to prevent adsorption of proteins. The antiadhesion capacity of the surface modified by polymers varies with the length of the chains, the inherent characteristics of the polymer, and the homogeneity and density of the polymer (Liu L. et al., 2014; Chen et al., 2021). The hydrophilic polymer polyethylene glycol (PEG), as one of the most widely used polymers, is often used for surface modification of implants because of its good antifouling properties, and its hydrophilic, flexible chains are essential to prevent protein and bacterial adhesion (Mas-Moruno et al., 2019; Pranjali et al., 2019; Buwalda et al., 2020). Skovdal et al. developed an ultra-dense poly(ethylene glycol) (udPEG) coating that was applied to Ti surfaces and demonstrated that the coating substantially reduced the adhesion of *Staphylococcus* epidermidis (S. epidermidis) both *in vivo* and *in vitro* and increased the efficacy of vancomycin. In a mice model, the coating effectively improved the therapeutic outcome of implant-associated infections in mice (Skovdal et al., 2018). Nishida et al. prepared a polymer coating containing the zwitterionic monomer, carboxymethyl betaine (CMB), on the Ti alloy surface, and the modified Ti alloy showed great potential as an implant surface modification material by strongly inhibiting protein adsorption and doubling the amount of calcium deposition (Nishida et al., 2017). Although the anti-adhesive property of PEG can favorably inhibit the adhesion of bacteria, it also reduces the tissue cells adhesion on the implant surface, which is detrimental for implant to integrate with the surrounding tissues, which can be addressed by the addition of RGD (one of the integrin-binding peptide sequences) (Mas-Moruno et al., 2019). Buxadera-Palomero et al. demonstrated by comparing three methods of preparing PEG coatings on Ti surfaces that the immobilization of RGD improved the adhesion of fibroblasts without affecting the adhesion inhibition of *Streptococcus* sanguinis (S. sanguinis) and *Lactobacillus* salivarius (L. salivarius) (Buxadera-Palomero et al., 2017).

In addition, there are polymers such as chitosan, polylactic acid, cellulose, and hydrogels that are often used as carriers of antimicrobial drugs and are used as modifying materials for implant surfaces. Chitosan, as a natural and sustainably sourced polysaccharide with antimicrobial properties that are effective against a wide range of target organisms and no toxicity, is widely used in tissue engineering, drug delivery and promoting healing. This may be related to the fact that chitosan bears cations that can interact with anions on the cell surface, inhibiting bacterial biosynthesis and interfering with their substance transport (Levengood and Zhang, 2014; Mishra et al., 2015; Palla-Rubio et al., 2019; Valverde et al., 2019; Rahayu et al., 2022). Chitin, chitosan are polymers with innate antimicrobial activities. Their abundance and multiple functional groups allow them to be modified to enhance their intrinsic antimicrobial activities. In addition, some polymers that are not antimicrobial have been functionalized with specific groups such as quaternary ammonium or guanidium groups and/or combined with Ag nanoparticles to produce antimicrobial properties (Haktaniyan and Bradley, 2022).

### 2.3 Antibiotics

Despite the proven disadvantages of antibiotics, such as microbial resistance, short drug duration of action, uncontrolled release and so on, they still have a significant role for management of infections, and applying antibiotics to implant surfaces is an effective strategy to improve their antimicrobial properties and reduce their side effects. For example, gentamicin (Baro et al., 2002; Laurent et al., 2008; Govindan and Girija, 2014; Diefenbeck et al., 2016; Albright et al., 2021; Nichol et al., 2021; Sang et al., 2021), vancomycin (Zhang L. et al., 2014), minocycline (Lv et al., 2014), tobramycin (Brohede et al., 2009; Zhou et al., 2018), amoxicillin, cefthiophene and other antibiotics have been explored to prepare an antibacterial coating. He et al. constructed an antimicrobial coating composed of gentamicin (GS) and polyacrylic acid (PAA) by layer-by-layer assembly (LBL) technique. The modified Ti exhibited outstanding antibacterial activity against *S. aureus* and *E. coli* and the release of gentamicin was slow and sustained during 11 days following an initial burst release in the first 24 h (He et al., 2020). In a mouse experiment, Stavrakis et al. demonstrated that the release of vancomycin from the PEG-PPS polymer coating was in a controlled manner and that *S. aureus* in implants containing vancomycin was reduced 139-fold and local concentration of the drug remained above the lowest inhibitory concentration of bacteria for 7 days postoperatively (Stavrakis et al., 2019). Wongsuwan et al. successfully developed a minocycline-loaded niosomes coating by thin-film hydration, which was applied to the implant surface by layer-by-layer spraying, and the modified implants showed satisfactory antibacterial efficacy against Porphyromonas gingivalis (P. gingivalis) and the drug release was maintained for more than a week (Wongsuwan et al., 2020). The application of various new materials and technologies has shown promising applications by improving the antibacterial efficacy of antibiotics, extending the duration of drug action, and reducing the side effects associated with systemic administration, but there is no consensus on the best treatment modality for the prevention of peri-implant infections with antibiotic-laden coatings. In addition, there are concerns about toxicity and bacterial resistance, so further research is needed regarding their use in dental implants (Esteves et al., 2022).

### 2.4 Antimicrobial peptides

Antibiotics are prone to induce drug resistance, which greatly limits its application, the emergence of antimicrobial peptides (AMPs) has provided a new approach to solve this problem (Zhang E. et al., 2021). AMPs are a class of short amphiphilic peptides having broad-spectrum antimicrobial properties, which are positively charged and can bind negatively charged bacteria by electrostatic attraction and interact with their cell membranes, leading to bacterial death (Lozeau et al., 2018; Sun et al., 2018). Studies have shown that AMPs can also enter the cytoplasm to act on intracellular targets (DNA, RNA, proteins, etc.), killing bacteria from the inside and affecting gene expression (Geng et al., 2018; Wang and Tang, 2019). Because of their specific antibacterial mechanism, the bacteria are hardly to develop drug resistance. Researchers have used different methods to coat antimicrobial peptides on the implant surface to prevent bacterial colonization, such as adsorption, binding, electrospinning, and chemical binding (Garaicoa et al., 2020; Tiwari et al., 2021 *)*. Yazici et al. designed an engineered chimeric peptide that coated peptide Tet127 on the microporous calcium phosphate on the surface of Ti nanotubes by self-assembly, and implants with the coating significantly reduced the adhesion of S. mutans and *E. coli*, and showed good osteointegration properties (Yazici et al., 2019). Cheng et al. prepared a new composite coating on the surface of smooth Ti containing the antimicrobial peptide Nal-P-113 and graphene oxide, which exhibited slow and sustained drug release *in vitro* and showed good antimicrobial performance against both P. gingivalis and S. mutans with no significant cytotoxicity against human gingival fibroblasts (Cheng et al., 2022). In a recent study, Fischer et al. coimmobilized the laminin-derived peptide LamLG3 and the antimicrobial peptide GL13K on the Ti surface, which exhibited antibiofilm activity against *Streptococcus* gordonii (S. gordonii) and was able to promote hemidesmosome formation, proliferation, and mechanical attachment of orally derived keratinocytes, while having no significant effect on fibroblast proliferation, showing the potential to reduce dental implant infection and failure rate (Fischer et al., 2020). In addition, Nie et al. found that the immobilization of KR-12 peptide on Ti surface using PEG as spacer showed better bactericidal efficiency than the surface without spacer (Nie et al., 2017). Therefore suitable spacers, properties of AMPs such as density, flexibility, *etc.*, are factors to be considered when we further optimize the antimicrobial effect of AMP-functionalized surfaces. In addition, the view that bacteria are unlikely to have evolved resistance to AMPs is increasingly being questioned due to their inherent survival strategies and evolutionary pressure. The relatively short history of their use has also meant that their clinical use has been inadequate and the mechanisms of their resistance have been poorly studied. It is possible for bacteria to develop resistance through mechanisms such as inactivation of AMPs, restrict access to AMPs, removal of AMPs, biofilm formation, *etc.* However, there is a wide variety of AMPs, and their functions are highly specific and synergistic. Accordingly, the acquisition of resistance by pathogenic bacteria is subject to evolutionary constraints, such as functional compatibility and fitness trade-offs. Therefore, we should conduct more research on the mechanisms by which bacteria acquire resistance to AMPs and their evolutionary constraints, and find techniques to exploit these constraints, such as applying AMP cocktails to minimize the efficacy of resistance selection or incorporating nanomaterials to maximize the cost of AMP resistance (Chen et al., 2022).

### 2.5 Chlorhexidine and other organic coatings

As a widely used antibacterial drug, chlorhexidine (CHX) has excellent antibacterial ability against both Gram-negative and Gram-positive bacteria and is generally applied for local disinfection and cleaning during oral care and oral surgery (Zheng et al., 2022). The cationic CHX molecule attracts to the surface of negatively charged bacterial cells and interacts with the cell membrane to disrupt its integrity, causing the cell contents to flow out, thus exerting its bacteria-inhibiting effects. As its concentration increases, it exerts a bactericidal effect by forming complexes with phosphorylated compounds, such as adenosine triphosphate and nucleic acids, which cause cytoplasmic coagulation and precipitation. In addition, the cationic character of the CHX molecule allows it to adhere to most negatively charged oral surfaces and interfere with the adhesion of bacteria (Poppolo Deus and Ouanounou, 2022). Garner et al. developed a CHX-hexametaphosphate coating for Ti surfaces and investigated its antimicrobial properties using a multispecies biofilm model, and determined its cytocompatibility with human mesenchymal stem cells (MSCs). The coating showed a significant reduction in multispecies biofilm formation within 72 h and had excellent cytocompatibility, allowing MSCs to perform their functions and differentiate into osteoblasts normally (Garner et al., 2021). Matos et al. prepared multifunctional CHX-doped thin films on pure Ti using the glow discharge plasma approach, where the release of CHX peaked at day 8 and maintained a slow release for 22 days. The modified surface exhibited significant inhibition of S. sanguinis biofilm growth and had good cytocompatibility (Matos et al., 2020).

In addition, some other organic antimicrobial agents have also shown great clinical value, such as totarol, rhamnolipids, chlorophenol, polyhexamethylene biguanidine, *etc.* Xu et al. used totarol, a natural antimicrobial agent, as a coating on Ti surfaces and silicon wafers, which showed that the totarol coating produced an efficient killing and inhibiting effect on S. gordonii and mixed oral bacterial biofilms. Its anti-adhesive and biofilm inhibitory effects persisted after 24 days of saliva incubation (Xu et al., 2020). Tambone et al. adsorbed rhamnolipids onto the Ti surface by physical adsorption and demonstrated that the coating had good antibacterial activity against *S. aureus* and S. epidermidis for up to 72 h, and the coated Ti samples showed no cytotoxicity (Tambone et al., 2021). These organic antimicrobial agents exerted antibacterial effect while avoiding the development of antibiotic resistance, and are promising agents for reducing the adhesion of bacteria and the formation of biofilms on Ti surfaces.

### 2.6 Natural product

The problem of increasing bacterial resistance to conventional antibiotics has prompted the search for new antimicrobial products. In recent years, there has been a growing interest in natural antimicrobial products that can effectively inhibit bacterial colonization and biofilm formation and are less likely to cause the development of bacterial resistance, making them promising alternatives or supplements to current synthetic antibiotics. For example, Fernández-Babiano et al. demonstrated that Carvacrol was able to exert a favorable antimicrobial effect against S. sanguinis and S. mutans, reducing the formation of biofilm (Fernández-Babiano et al., 2022). Alonso-Español et al. demonstrated the inhibitory effect of Curcumin and Xanthohumol on multi-species biofilms on the surface of implants containing *Actinomyces* naeslundii (A. naeslundii), *Streptococcus* oralis (S. oralis), Veillonella parvula (V. parvula), *Fusobacterium* nucleatum (F. nucleatum), Aggregatibacter actinomycetemcomitans (A. actinomycetemcomitans) and P. gingivalis (Alonso-Español et al., 2023). In addition, natural products such as propoli (Navarro-Pérez et al., 2021), Colocasia antiquorum var. esculenta (CA) varnish (Shin et al., 2022), nasturtium officinale extract (Tabesh et al., 2022) and Rosmarinus officinalis extract (Günther et al., 2022) also have been studied for their antimicrobial effects. Some natural products have been explored for use as antimicrobial coatings on implant surfaces to prevent the development of peri-implantitis. Córdoba et al. functionalized Ti surfaces by covalently grafting phytate (IP6) directly onto Ti surfaces through Ti-O-P bonds without the use of cross-linker molecules. The obtained Ti-IP6 surface significantly reduced the adhesion of S. sanguinis and induced the gene expression of osteogenic markers, suggesting that it has a good antibacterial property and osteogenic potential (Córdoba et al., 2016). Gomez-Florit et al. successfully grafted quercitrin onto Ti surface, prepared quercitrin-nanocoating, and explored its antimicrobial properties with S. mutans, and tested its anti-inflammatory properties and potential to promote soft tissue regeneration with human gingival fibroblasts. The results showed that quercitrin-nanocoating significantly reduced the initial adhesion of S. mutans and biofilm formation, while increasing the attachment of human gingival fibroblasts and decreasing the expression of related inflammatory factors, indicating the value of the coating in the promotion of soft tissue integration and the prevention of peri-implantitis (Gomez-Florit et al., 2016). Natural antimicrobial products have outstanding antimicrobial properties and various efficacies, showing good application prospects, but most of them have complex compositions and various targets, so the dosage and specific mechanisms of various natural products need to be further explored.

### 2.7 Trigger-responsive antimicrobial coatings

**FIGURE 3:**
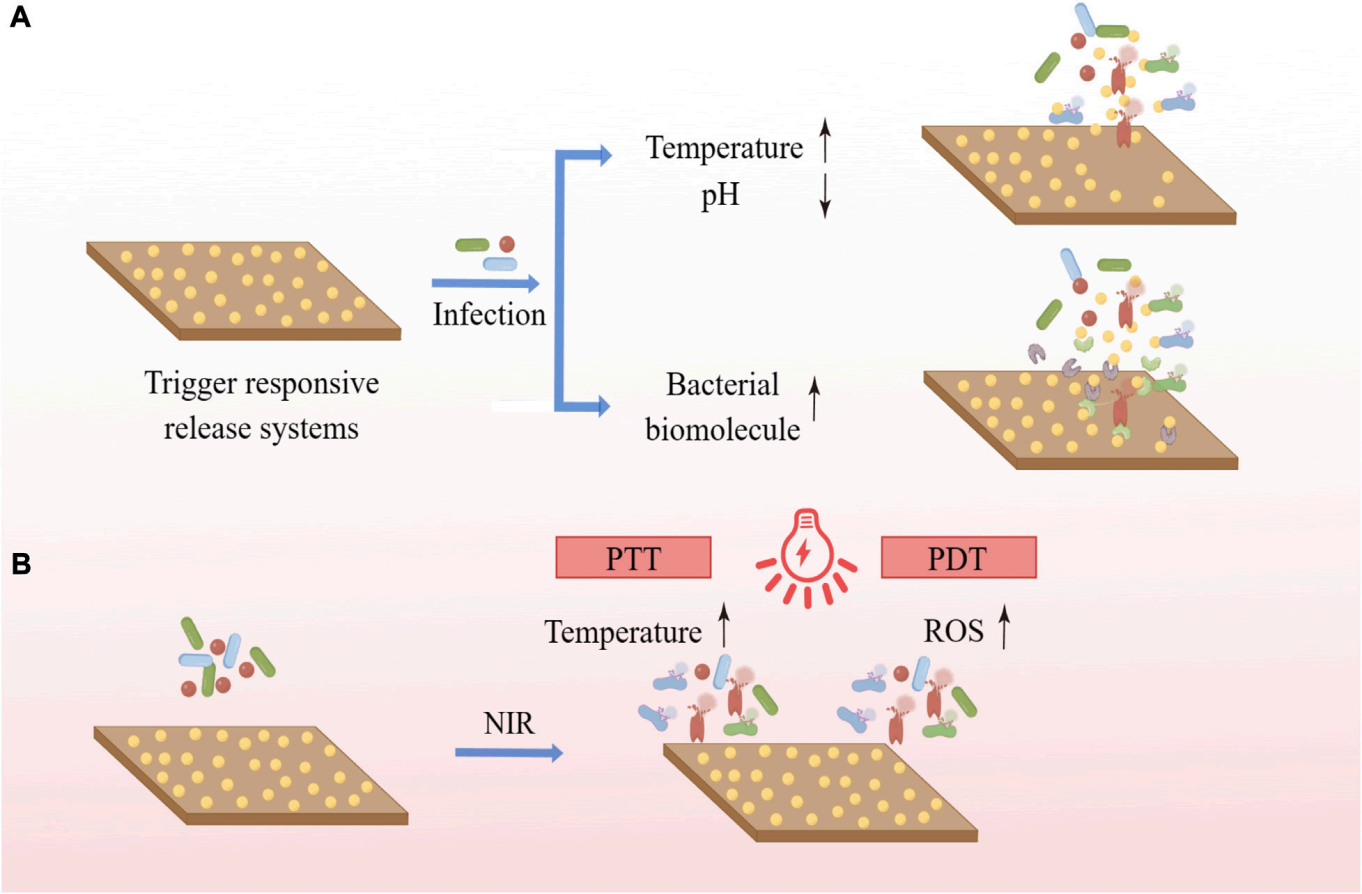
**(A)** Trigger responsive coatings respond to changes in the local microenvironment or biomolecule concentration caused by bacterial infection to initiate the release of the cargo. **(B)** PTT uses photothermal agents to convert light energy into heat, and PDT uses photosensitizers to trigger photochemical reactions such as the production of ROS to exert antimicrobial effects.

The application of a trigger responsive release systems allows the release of the drug to occur only when needed, greatly increasing the drug utilization and enhancing the antimicrobial activity and specificity of the coating (Skovdal et al., 2018). Such coatings can initiate the release of drugs by responding to variations of the local microenvironment or biomolecular concentration induced by bacterial infection (Figure 3). For instance, bacterial infection causes changes such as decrease in pH of the local microenvironment, increase in temperature, and so on. Lee et al. prepared a pH-responsive cinnamaldehyde-TiO_2_ nanotube coating (TNT-CIN) by silylation, hydroxylation, anodic oxidation, and Schiff base reaction, which showed better drug release in low pH condition, good anti-inflammatory, osteogenic, and anti-bacterial abilities, and the viability and number of P. gingivalis and S. mutans on the surface were significantly lower than those of uncoated TNT (Lee et al., 2022). Choi et al. used copolymers made up of 2-hydroxyethyl methacrylate and diethylene glycol methyl ether methacrylate to prepare a multi-layered temperature-responsive polymer brush (MLTRPB) coating on a Ti surface, which triggered a significant release of antibiotics from the coating at 38°C–40 °C, the local temperature that may be reached during infection. The coating showed good antimicrobial efficacy in both *in vitro* and *in vivo* experiments (Choi et al., 2022). In addition, certain specific enzymes released by bacteria during infection can also act as triggers. Li et al. loaded ciprofloxacin (CIP) into mesoporous polydopamine (MPDA) nanoparticles (MDPA@CIP), anchored them to the Ti surface, and covered them with a hyaluronic acid-catechol (HAc) coating. Bacterial hyaluronidase secreted by bacteria can accelerate the degradation of HAc, which allows the on-demand release of antimicrobial drugs at the site of infection and enables functional Ti to exhibit good antimicrobial ability (Li D. et al., 2023). Materials responsive to bacterial metabolites are beneficial to adaptive antimicrobial systems and precision medicine, allowing drug exposure or release to occur only when and where it is needed and reducing the misuse of antimicrobials and the development of bacterial resistance. Changes in the microenvironment of infection and the concentration of biomolecules can also help us detect bacterial infections and monitor bacterial growth, reflecting the extent of the infection in time to better prevent the occurrence of disease. Therefore, a stimulus-responsive antimicrobial system with both monitoring and therapeutic functions is worthy of further research (Wang et al., 2022).

**FIGURE 4:**
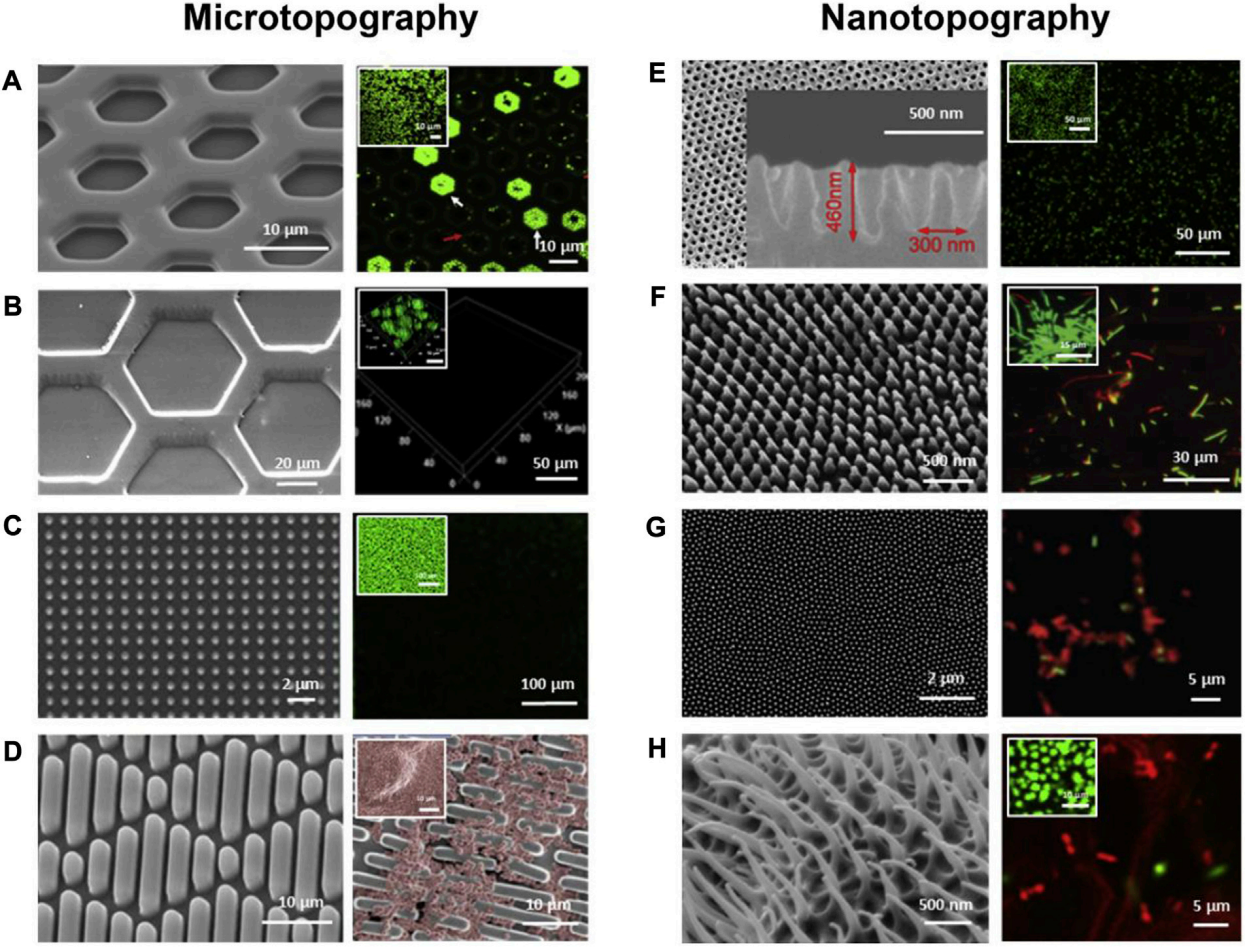
SEM images (left) and fluorescent microscopic images (right) of bacterial attachment on micron- and nano-scale topographies and the small images show cell attachment on flat control surfaces. **(A)** Hexagonal PDMS pits; **(B)** Hexagonal PDMS pillars; **(C)** Micropillars; **(D)** Sharklet™; **(E)** Parabolic nanostructures; **(F)** Nanopillars; **(G)** Cicada wings; **(H)** Gecko skins. Green represents live cells **(A–C**, **E–H)** and red represents dead cells **(E–H)**. The red color shows the live cells **(D)**. Reproduced with permission from (Lee et al., 2021). Copyright (2020) Elsevier.

Near infrared (NIR) irradiation-based photothermal therapy (PTT) and photodynamic therapy (PDT) are increasingly of interest due to their low invasiveness and good region-selectivity, having been extensively investigated in areas ranging from cancer treatment to infection relief. PTT uses photothermal agents to convert light energy into heat, while PDT uses photosensitizers to trigger photochemical reactions such as the production of ROS for treatment (Ghimire and Song, 2021) (Figure 4). In the presence of light, polydopamine (PDA), MoSe_2_, MnO_2_, TiO_2_, ZnO, IR780 phosphorus, graphene oxide and indocyanine green (ICG) can increase local temperature and/or produce ROS, which can kill bacteria (Kubiak et al., 2021). Chai et al. synthesized molybdenum diselenide (MoSe_2_) on the surface of porous Ti dioxide layers prepared by micro-arc oxidation on Ti implants using a hydrothermal method, electrostatically bonding chitosan (CHI) to MoSe_2_ nanosheets to improve their biocompatibility. The TiO_2_ coating introduced with MoSe_2_ has good photothermal and photodynamic capabilities and shows excellent antibacterial properties under 808 nm NIR light irradiation due to the synergistic effect of high temperature and ROS (Chai et al., 2021). The photothermal and photodynamic antibacterial strategies can exert good antibacterial effects and do not cause antibiotic resistance. At the same time, it can precisely control the irradiation site, time and dose, and is easy to operate without being affected by the surrounding environment. However, the penetration of light into soft and hard tissues is limited and prolonged infrared irradiation is harmful, while short-term temperature increase and ROS production are not sufficient to effectively cure recurrent bacterial infections, which is why its application on implant surfaces requires further research.

### 2.8 Methods of constructing coatings

Coatings are formed by the attachment or diffusion of antimicrobial agents or bioactive elements to the substrate, or by changes in surface properties to improve the antimicrobial as well as soft/hard tissue integration capabilities of the surface. A variety of ways have been developed to construct implant surface coatings, the earliest of which was plasma spraying (Claes et al., 1976), a thermal spraying coating process that uses high temperatures to melt and spray powders on surfaces to produce a high-quality coating. People usually use powders such as Ti, hydroxyapatite, *etc.*, for surface modification of implants to improve their osseointegration. In order to enhance the antimicrobial activity, various antibiotics have been used in combination with calcium phosphate for surface modification of implants, such as cephalothin (Ke et al., 2017), streptomycin (Li et al., 2015), gentamicin (Govindan and Girija, 2014), and tetracycline (Ratier et al., 2001). However, extremely high temperatures make it difficult to incorporate organic compounds into the coatings in the plasma spraying technique, and the antibiotics are released too quickly to maintain antimicrobial capacity. Therefore, attempts have been made to introduce metal ions and nanoparticles with antimicrobial properties into the coatings, such as Ag (Ruan et al., 2009; Ke et al., 2019), Zn (Vu et al., 2019), *etc.* These coatings have shown good antimicrobial capabilities, but Ti alloys with plasma-sprayed hydroxyapatite have weak bonding strengths, and doubtful long-term stability after implantation, so better materials and techniques still need to be explored. In addition, Metal Ions Implantation (MII) is also a well-researched technique. The antimicrobial effects of many metal elements have been known, such as Ag (Tan et al., 2023), Cu(Wu L. et al., 2022), Zn (Li X. et al., 2019), cerium, *etc.*, which have been loaded into implants to improve the antimicrobial properties of implants. In addition to the improvement in antimicrobial properties, the technique also provides a significant improvement in the corrosion and abrasion resistance of the modified surface, which has led to its widespread application. The sol-gel method has attracted attention for its ability to produce coatings with high purity and homogeneity with relatively low temperatures, and it can be used to prepare a variety of metal oxide materials, as well as a number of coatings, films, and fibers that are difficult to melt with conventional processes, which is a commonly used technique for deposition of HA films on implants (Chai et al., 1998). Good antimicrobial properties can be obtained by replacing the calcium ions of HA with metal ions that have antimicrobial effects or replacing its hydroxyl ions with fluoride ions in the sol-gel method (Batebi et al., 2018). In addition, micro-arc oxidation (MAO), sputtering deposition, and Layer-by-Layer (LBL) self-assembly have also been used to construct surface coatings to improve the antimicrobial and osteointegration capabilities of implants.

## 3 Surface topography

Various approaches that rely on the addition of chemical agents to the surface face two challenges: safety and long-term antimicrobial efficacy. Therefore, more attention has been paid to modifying the physical properties of the implant surface to improve the antimicrobial performance. Because it does not consume drugs, physical modification of the surface allows for more long-term effectiveness, less adverse effects on surrounding tissues, and does not need to worry about causing drug resistance of bacteria, so it is considered to be a more promising approach than chemical modification (Wu et al., 2018). Surface topography, an important parameter of the physical properties of the surface, includes surface roughness as well as profile shape (Ardhani et al., 2022). The effects of implant surface roughness and microscopic profile shape on bacterial adhesion and activity have been extensively studied.

### 3.1 Surface roughness

The most commonly used parameters to describe the characteristics of surface topography are the root-mean-square surface roughness (Rrms) as well as average surface roughness (Ra), which represents the root-mean-square and average deviation of height values from the mean line, respectively (Wu et al., 2018). A slightly rough surface is more conducive to osseointegration, and to obtain better surface properties, the implant surface is usually treated to improve cell adhesion and reduce bacterial adhesion. Commonly used methods include sandblasting, acid etching, large particle sandblasting acid etching (SLA), anodic oxidation, and fluoride treatment (Yeo, 2014; Han et al., 2017). Of these methods, large particle sandblasting acid etching is one of the most popular ways, which can increase surface roughness, enlarging the surface area for osteoblasts to attach, which is beneficial to osseointegration and interfacial stress distribution (Wang et al., 2020). However, an increasing in roughness will also increase the adhesion of bacteria while improving osseointegration because the rough surface provides more attachment sites and “shelters” for bacteria to resist shear forces (Robinson et al., 1996; Teughels et al., 2006). On the contrary, mirror polished surfaces have good resistance to bacterial adhesion and biofilm formation, yet such flat surfaces are detrimental to the adhesion of surrounding tissues (Guo et al., 2021). However, some researchers have obtained different results. In one study, Pacha-Olivenza et al. investigated the adhesion and proliferation of human gingival fibroblasts and bacterial strains: A. actinomycetemcomitans, S. sanguinis and S. mutans on the surface of Ti disks with different surface treatments: machined (Mach), nitrided (TiN), lightly acid etched (AEn), strongly acid etched (AEt) and sandblasted/acid etched (SB + AE). The results showed that smooth surfaces are more favorable for adhesion and proliferation of fibroblasts, and the overall race between cells and bacteria for surfaces also favored smoother surfaces (Mach, TiN and AEn) compared to rougher surfaces (Aet and SB + AE) (Pacha-Olivenza et al., 2019). In addition, there are also some researchers suggesting that increased surface roughness does not affect or even inhibit bacterial adhesion (Rizzello et al., 2011; Bagherifard et al., 2015; Liu et al., 2016; Lüdecke et al., 2016). The contradictory results may be due to the differences in materials and bacterial strains used, and the lack of a comprehensive analysis of the distribution of surface microtopography, profile shape, *etc.* Ra and Rrms can only describe the height of surface features, but not their lateral size, shape, spacing, *etc.* Surfaces with the same Ra and Rrms values may have different surface features, and these parameters can have a significant impact on bacterial adhesion, so we need more parameters for comprehensive analysis of surface morphology data. Stout et al. proposed a set of 14 roughness parameters for the comprehensive analysis of surfaces, called “Birmingham 14”. Crawford et al. suggested that at least RMS surface roughness (Sq), summit density (Sds), developed area ratio (Sdr) is required to study the relationship between surface roughness and bacterial adhesion (Crawford et al., 2012). However, more accurate and concise schemes for characterizing surface topography still need to be further explored. In addition, the interaction between bacteria and surfaces changes over time. Han et al. obtained different surface roughnesses on zirconia surfaces by different surface treatments with Ra values of: 0.17 ± 0.03 µm (untreated), 0.56 ± 0.05 µm (grit-blasting), 1.47 ± 0.04 µm (HF-etching), 1.48 ± 0.05 µm (grit-blasting followed by HF-etching). It is interesting to note that the highest number of P. gingivalis was found on the two surfaces with the highest Ra value (the last two groups) at 24 h. However, after 72 h, the amount of biofilm on the two surfaces with the most bacteria became the lowest. The authors speculate that this change may be related to their high hydrophilicity and low surface energy. Thus, the effect of surface roughness on the adhesion of bacteria may be diminished after biofilm maturation (Han et al., 2017).

In general, it is believed that bacterial adhesion and biofilm formation are more likely to occur when the surface roughness exceeds the Ra threshold of 0.2 µm, whereas Ra values below 0.2 µm do not lead to a further decrease in bacterial adhesion (Bollen et al., 1997). Nano roughness has been proposed to be suitable for preventing the adhesion of microorganisms. The reason is that most bacteria, as well as P. gingivalis, have a length of about 1.51 µm and a diameter of about 1 µm. On surfaces with topographical features at the micrometric scale, which is similar in size to bacterial cells, cells tend to position themselves to maximize their contact area with the surface, whereas on surfaces with topographical features at the submicrometric and nanometric ranges, which are much smaller than bacterial cells, cells inhibit adhesion by reducing the contact area between the cells and the surface (Amoroso et al., 2006; Feng et al., 2015; Mukaddam et al., 2021). Various studies have revealed that micro/nanostructures are not of particularly high or low roughness, having different roughness levels according to the surface patterns (Huang et al., 2017; Kunrath et al., 2020). The same is true for surface energy, wettability, *etc.* There is a study in which the water contact angle on a microsurface was found to increase with the increase of roughness until a plateau stage is reached (Giljean et al., 2011). These can have an impact on the interaction between bacteria and the surface, and thus on the adhesion of bacteria to the surface.

### 3.2 Patterned surface topography

The surfaces of various plants and animals in nature have natural anti-fouling and antibacterial properties, such as shark skin (Sakamoto et al., 2014; Chien et al., 2020; Chien et al., 2021; Zhang H. et al., 2023), worm skin (Hayes et al., 2016), lotus leaves (Latthe et al., 2014; Jiang et al., 2020), butterfly wings (Bixler and Bhushan, 2014), cicada wings (Hazell et al., 2018; Shahali et al., 2019; Mo et al., 2020; Liu et al., 2022), dragonfly wings (Bhadra et al., 2015; Mathew et al., 2023), *etc.*, which suggest that specific nano/micron-scale structures have antibacterial effects. Inspired by nature, various specific patterns have been prepared on artificial materials to study the interaction between bacteria and surfaces as well as to design surfaces with antimicrobial properties. The animal topography most widely reported for biomedical surfaces modification is sharkskin, where the microscopic shape of the surface and the distribution of denticles (diamond-shaped scales that cover the animal’s outer surface) give it very outstanding anti-fouling and self-cleaning properties (Liu et al., 2020). Surfaces with the ability to prevent bacterial adhesion have been developed mimicking shark skin with topographic features of rectangular shape with a height of 3 μm, width of 2 μm, and length of 4–16 μm, arranged periodically in a diamond shape, with a distance of 2 µm between adjacent features (Damodaran and Murthy, 2016). The distribution, shape, and size of topographic patterns affect bacterial adhesion. Vadillo-Rodríguez et al. prepared surfaces with topographical features having different shapes and sizes using polydimethylsiloxane (PDMS), which include protruding and receding circular and square features and parallel channels and ridges, with micrometer-scale lateral dimensions (spacing and length/width) and nanoscale heights, to evaluate the effect of nanoscale roughness on bacterial adhesion and biofilm formation. The results showed that all surface patterns significantly inhibited bacterial adhesion and biofilm formation, and that the bacterial cells actively chose their initial locations for adhesion, favoring those geometrical locations that maximized the cell-surface contact points/areas, including square corners and convex walls of recessed surface features. This study suggests that we can control the initial location of attachment of adherent cells by the particular geometry of the features (Vadillo-Rodríguez et al., 2018). Many topographic features have been shown to have an inhibitory effect on the formation of biofilms, such as honeycombs (Vasudevan et al., 2014; Yang et al., 2015; Gu et al., 2016; Liao et al., 2021; Li S. et al., 2023), irregular micro pits (Jeong et al., 2017), line patterns (Lee and Pascall, 2018; Wu et al., 2020; Damiati et al., 2022; Sorzabal-Bellido et al., 2022), ridges (Zhang B. et al., 2014; Perera-Costa et al., 2014; Lu et al., 2016), cylindrical wells (Fontelo et al., 2020; Xiao et al., 2020), square pillars (Hou et al., 2011; Halder et al., 2014; Ge et al., 2015; Valle et al., 2015; Ge et al., 2019), hexagonal pillars (Chang et al., 2018; Pingle et al., 2018). Although the patterns, materials, and strains used vary, most patterns inhibit bacterial adherence more significantly at a smaller size.

### 3.3 Micron- and nano-scale topographies used for implants

Various micro/nanostructure topographies have been developed for surface modification of implants to enhance their antimicrobial properties (Figure 4). The influence of surface topography on the bacterial attachment is largely dependent on the size. Generally speaking, micron-scale topographies are not bactericidal, but can influence bacteria-surface interactions to prevent the adhesion of bacteria and impede the formation of biofilms. In contrast, many nanoscale topographies can disrupt bacterial cell membranes directly to provide a bactericidal action. For instance, nanopillars work similarly to a "bed of nails" that disrupt the cell membrane of bacteria in contact with them (Khalid et al., 2020; Lee et al., 2021).

Titanium nanotubes (TNTs) are highly ordered nanotubular structures fabricated on Ti surfaces, which are easy to fabricate, can be obtained in specified sizes by changing their design parameters, have good biocompatibility and antibacterial properties, and are widely used for surface modification of Ti. Camargo et al. prepared Ti dioxide nanotubes with diameters of 100 nm and 150 nm by anodic oxidation of Ti oxide. After 30 days of bacterial incubation, the structured surface of Ti dioxide nanotubes showed significant resistance to P. gingivalis and dense spirochetes of dental tartar (Camargo et al., 2021). Peng et al. prepared arrays of Ti oxide nanotubes with diameters of 30 nm and 80 nm, respectively, on Ti substrates by a two-step anodic oxidation method, and compared with the control group, it was observed that the surface roughness of Ti dioxide nanotubes increased, the water contact angle decreased, and the adhesion and colonization ability of S. epidermidis on its surface was significantly reduced (Peng et al., 2013). The antibacterial mechanism of TNTs may include: mutual repulsion of negative charge of TNTs and negative bacterial surface charge; stretching of bacterial membranes by nanotubular structures leading to cell membrane rupture and bacterial death; and high surface roughness of nanostructures increasing surface hydrophilicity and preventing attachment of hydrophobic bacteria (Li et al., 2019e). There are also different nanopatterns that are available to reduce bacterial attachment besides nanotubes. Kim et al. generated a surface consisting of 50–100 µm micropores and 25–30 nm nanopores by nitriding and anodic oxidation on the surface of Ti discs, and this surface significantly inhibited the adhesion of P. gingivalis and S. mutans, showing excellent antibacterial activity (Kim et al., 2021). In addition, many other nanostructured surfaces such as nanogrooves (Ferraris et al., 2017), nanopillars (Yi et al., 2018; Wang S. et al., 2023), and biomimetic micro/nanostructured surfaces (Elbourne et al., 2017; Ye et al., 2019; Mo et al., 2020; Zhang Y. et al., 2023) have been shown to have good antibacterial properties.

In addition to their antimicrobial effects, nanostructures can also influence the interaction of the surface with cells and tissues. It has been shown that some nanostructures also have the ability to facilitate the migration, adhesion, and growth of cells. A study by Wu et al. found that Ti dioxide nanotube arrays can affect the type of adsorbed proteins and change their conformation; improve adhesion of macrophages and induce polarization T lymphocytes; promote repairment-related cells adhesion and filopodia formation; and induce osteogenic differentiation and blood vessel formation (Wu B. et al., 2022). Ferraris et al. showed that the nanogrooves can drive the alignment of gingival fibroblasts, while the keratin nanofibers deposited can increase cell adhesion and proliferation, through respectively a topographical and a chemical stimulus (Ferraris et al., 2017). Surfaces with host cell coverage are largely resistant to the colonization of bacteria because of the " race for surface " principle (Gristina, 1987). Mezey et al. also demonstrated in a previous study that mesenchymal stem cells (MSCs) possess inherent antibacterial properties (Mezey and Nemeth, 2015). Therefore, while focusing on their antimicrobial effect, we should also take into account the affinity of the surface topography for the host tissue and cells. Currently, the topography that prevents bacterial colonization and facilitates tissue integration has not been well studied, and more attention should be devoted to this aspect.

At the same time, there are a large number of studies using the combination of surface topography and antibacterial agents to obtain antifouling and antibacterial properties and inhibit the formation of biofilm. Dolid et al. prepared a surface with shark skin topography and coated the micropatterned surfaces with a peptide (DOPA-Phe(4F)-Phe(4F)-Arg-Gly-Asp) coating and tested the adhesion of *E. coli* and S. epidermidis under static conditions and in dynamic experiments, and the results showed that the micropatterned surfaces with peptide coatings in both cases had better anti-adhesive effects than the other three groups (smooth surfaces; smooth surfaces with coatings; micropatterned surfaces) (Dolid et al., 2020). In addition to biomedical surfaces, nanotubes, mesoporous structures, *etc.*, are also commonly used to load antimicrobial agents to promote the synergistic effect of surface topography and antimicrobial drugs. Nanotubes have the ability to load, store, and release bactericidal agents in addition to their excellent anti-microbial adhesion properties. Antibiotics, antimicrobial peptides, and metal nanoparticles can be combined with Ti nanotubes to exert good antimicrobial effects (Li et al., 2017; Arkusz et al., 2020; Mansoorianfar et al., 2020). Ti dioxide nanotubes doped with hydroxyapatite (HA), selenium (Se), and silver (Ag) compounds (Ag_2_SepTNT) were prepared on the surface of Ti alloy (Ti_6_Al_4_V) by Staats et al. The modified surface showed good antimicrobial effects against S. epidermidis (Staats et al., 2022). In addition, the surface topography design promotes the separation of bacterial cells and delays their biofilm formation, which can improve the susceptibility of bacteria to antibiotics and reduce their drug resistance (Khalid et al., 2020).

### 3.4 Methods for improving surface topography

Sandblasting and acid etching technology is one of the most commonly used surface modification methods in commercial implants and is considered a safe and effective treatment. Sandblasting involves the projection of particles of various diameters to the surface to remove surface contaminants and obtain a roughened surface that improves the strength between the implant and the bone, increasing the osseointegration. Acid etching is the process of treating a surface using a strong acid such as HCl, HNO3, or H2SO4 to obtain microscopic pits that improve cell adhesion and osseointegration. People usually perform acid etching after sandblasting, and the whole process is considered as the reference surface treatment, called sandblasting and large grit acid etching (SLA) (Accioni et al., 2022). The ideal surface can be obtained by adjusting the parameters such as the type and size of abrasive, as well as the type and concentration of acid, and the treated surfaces obtained a better osseointegration and the SLA-treated zirconia surfaces had less biofilm formation than the pure Ti surfaces (Roehling et al., 2017). Anodic Oxidation is also one of the more commonly used methods to improve the surface morphology of implants by preparing uniform nanotube structures on Ti surfaces (Shin and Lee, 2008). The obtained nanotube structures have higher roughness and hydrophilicity and increased surface area, which can improve soft and hard tissue integration and reduce bacterial adherence (Li et al., 2019d; Pan et al., 2021). In addition, Ti dioxide nanotubes have the ability to generate ROS via photocatalysis and to be used as carriers loaded with antimicrobial drugs to exert antimicrobial effects (Podporska-Carroll et al., 2015; Fathi et al., 2019). Laser ablation technology uses a laser source to melt metals to obtain micron and nanostructures, which has the advantage of being able to maintain mechanical properties and reduce the risk of contamination. The surface roughness of the treated Ti is altered and ideal osseointegration as well as antimicrobial capacity is achieved through its high hydrophobicity (Hu et al., 2018). Alkaline heat (AH) treatment and acid-alkali treatment are also used to form micro- and nanoscale rough surfaces on Ti surfaces, and both result in modified surfaces with good antimicrobial properties (Zhang and Liu, 2015; Zhong et al., 2022). In addition, the antimicrobial properties of biomimetic topography have received much attention in recent years, and a series of methods to generate biomimetic topography have been explored, such as metal assisted chemical etching, hydrothermal synthesis, nanoimprint lithography, electron beam lithography, *etc.*

## 4 Intrinsic antibacterial materials

Pure Ti or Ti alloy (Ti_6_Al_4_V) has always been a popular material for implants due to its good biocompatibility, outstanding mechanical properties and high resistance to corrosion. However, pure Ti lacks favorable antimicrobial properties whereas various surface modifications are rarely used clinically due to limitations in safety, coating degradation and drug resistance, which has led to the development of alternative materials for implants with better antimicrobial properties.

### 4.1 Alloys

The lack of antimicrobial properties of pure Ti and Ti_6_Al_4_V, the most popular materials used for implants, is a major disadvantage. Ag, Cu and Zn are often added as antimicrobial reagents to the implant surface by a variety of surface modification methods (e.g., magnetron sputtering, plasma spraying, and plasma immersion ion implantation) to improve the antimicrobial properties of the implant, but these surface materials are gradually depleted and do not have a long-term antimicrobial effect. As a result, antimicrobial alloys have been developed to replace antimicrobial coatings and to provide good antimicrobial properties throughout the implant rather than just on the surface.

Cu and Ag are the most commonly used metallic elements added into alloys as antimicrobial agents. Cu which is an essential trace element for living organisms has become a widely used alloying element because of its outstanding properties such as promoting fracture healing, being antibacterial and protecting the cardiovascular system (Ren et al., 2015). As an element with strong biological activity, Cu has powerful bactericidal and cytotoxic effects. Liu et al. found that when the submerged concentration of Cu was higher than 0.036 mg/L (much lower than the daily intake recommended by the World Health Organization), the antibacterial rate could reach more than 99% (Liu J. et al., 2014). This goes some way to demonstrating the safety of Ti alloys containing Cu. In a recent study, Yang et al. developed Ti-Cu sintered alloys (3 wt% and 5 wt% Cu, i.e., Ti-3Cu and Ti-5Cu) and evaluated their antimicrobial activity against S. mutans and P. gingivalis. It was revealed that the Ti-Cu alloys showed time-dependent antibacterial activity against both bacteria. The antimicrobial activity of the Ti-Cu alloy was correlated with the release of Cu^2+^ and the production of ROS. At different time points, Ti-5Cu released higher concentrations of Cu^2+^ than Ti-3Cu and also showed better antibacterial activity. 70% and 57% antibacterial activity against P. gingivalis and 78% and 63% antibacterial activity against S. mutans at 72h for Ti-5Cu and Ti-3Cu respectively (Yang et al., 2023). Zhang et al. evaluated the antibacterial and osteogenic properties of a series of hydrogen fluoride (HF) etching + anodised Ti-Cu alloy with different Cu contents (3 wt%, 5 wt% and 7 wt% Cu). The Ti-Cu alloys were shown to have strong antimicrobial properties and good biocompatibility and osteogenic ability. It was also concluded that the antibacterial rate was higher and more stable when the Cu content was ≥5% (Zhang W. et al., 2021). In the case of Ti-Cu alloys, however, the antimicrobial ability did not always correlate positively with the amount of Cu. It is also related to the form of Cu present in the alloy, and the amount of Ti_2_Cu phase in the alloy is closely related to the antibacterial strength of the alloy (Fowler et al., 2019). Therefore, researchers can increase the content of Ti_2_Cu to improve its antibacterial ability by different treatments rather than continuously increasing the content of Cu in the alloy. In addition to Ti-Cu alloys, Ti-6Al-4V-xCu alloys, Ti-Zr-Cu alloys and others have also been investigated. For example, Xu et al. suggested that Ti-6Al-4V-5Cu has superior corrosion resistance and cell viability than Ti-6Al-4V, as well as exhibiting outstanding antibacterial ability, the antimicrobial activity of which may be associated with the bactericidal effect of the large amount of Ti_2_Cu precipitation (Xu et al., 2021).

Ag which has good antimicrobial properties and the ability to resist the formation of biofilm has been used for thousands of years as an antibacterial agent. Similar to Ti-Cu alloys, the antimicrobial ability of Ti-Ag alloys is also correlated closely with the amount of Ag. For example, Maharubin et al. developed Ti-Ag alloys containing various Ag contents (0.5 wt%-2 wt% Ag) and evaluated the antimicrobial activity against Gram-negative (*P. aeruginosa*) and Gram-positive (*S. aureus*) strains. 92% biofilm inhibition was achieved for *P. aeruginosa* and 93% for *S. aureus* after 3 h on the 1% Ag alloy. For the 1.5% Ag alloy, the inhibition rates were 99.96% and 99.7%, respectively, while the continued increase in Ag content did not result in a significant increase in antibacterial performance. Therefore, the antibacterial ability enhanced with increasing Ag content, but was not linearly correlated and higher concentrations of Ag may be harmful to mammalian cells (Maharubin et al., 2019). Nakajo et al. also demonstrated that Ti-Ag alloys do not affect bacteria when the Ag content is higher than 30 wt% Ag (Nakajo et al., 2014). Similar to Ti-Cu alloys, the antimicrobial ability of Ti-Ag alloys is also dependent on the content of their Ti_2_Ag phase (Chen et al., 2016). In addition, good antibacterial activity has been obtained by adding Ag to the Ti-Nb alloy Ti-Nb-Zr (Ou et al., 2017; Cai et al., 2021). They suggest that the deposition of the Ag-rich phase is the main reason for its antimicrobial properties, but the presence of the Ti_2_Ag phase increases the Young’s modulus of the material, which is detrimental to the matching of Young’s modulus between the human skeleton and the metallic material, so how to balance Young’s modulus and antimicrobial efficacy is also a challenge for the development of antimicrobial Ti alloys.

### 4.2 Ceramics

Ceramics are excellent candidates for implant materials owing to their biocompatibility, wear resistance and chemical stability, as well as their excellent aesthetic properties. A variety of ceramic materials have been studied to explore their potential as implant materials, including alumina (Flamant et al., 2016), tricalcium phosphate (TCP) (Kawamura et al., 2003), hydroxyapatite (HA) (Ichikawa et al., 1998), silicon nitride (Zhou et al., 2021), zirconium oxide (Dantas et al., 2021), bioglass (Fu et al., 2018), *etc.*

As a bioceramic, zirconia is a promising material for implants with a wide range of applications in dentistry. There are already zirconia dental implants on the market, however, zirconia dental implants are still lacking in research to be a popular choice. It has been found that zirconia implants exhibit a repulsive effect on bacteria and inhibit biofilm formation due to hydrophobicity and electrical conductivity, therefore, zirconia is more resistant to bacterial adhesion and biofilm formation than Ti (Karygianni et al., 2013; Nascimento et al., 2016; Hanawa, 2020; Oda et al., 2020). Roehling et al. compared the formation of biofilms on the surfaces of pure Ti and zirconia using three-species biofilm of P. gingivalis, F. nucleatum, S. sanguinis and human plaque samples in an anaerobic flow chamber model, demonstrating that after 72 h of incubation, the thickness of the three-species biofilm and human plaque on the surface of zirconia implants was significantly lower than that of pure Ti, but the quality and metabolism of the biofilms did not show significantly difference (Roehling et al., 2017). Another *in vivo* study also demonstrated that during experimental plaque accumulation, the total number of bacteria around the Ti implants and the counts of Tannerella forsythia (T. forsythia) and Prevotella intermedia (P. intermedia) were higher than in the zirconia implants and produced a stronger inflammatory response (Clever et al., 2019). However, some studies have come to a different conclusion, for example, when Siddiqui et al. evaluated the growth of oral early colonising bacteria and mammalian host cells on commercial pure Ti and zirconium oxide surfaces, in which they found no significant difference in the bacterial counts of Streptococcal strains adhering to commercial pure Ti and zirconium oxide surfaces after 1 or 3 days (Siddiqui et al., 2019). The reasons for this variation may be as diverse as the experimental set-up, the strain, the surface treatment, *etc.* Therefore, more research is still needed on the performance of zirconia implants. In addition, silicon nitride showed better osseointegration and anti-infection properties than Ti and polyether ether ketone (PEEK) in experiments conducted by Webster et al. in rats (Webster et al., 2012). Combined with the remarkable mechanical properties, silicon nitride is considered to be an attractive ceramic implant material (Rahaman and Xiao, 2018). Ceramic materials are of great interest for their chemical stability, biocompatibility, mechanical properties and antimicrobial properties, but certain properties such as brittleness and low ductility significantly limit their clinical applications. Therefore, researchers should focus on improving the physicochemical properties of these materials while improving their antimicrobial properties through various modification methods.

## 5 Summaries and perspectives

With the increasing number of patients receiving dental implants, peri-implant disease has become a major concern. While exploring and optimizing the treatment of peri-implant disease, efforts have been made to enhance the antibacterial ability of the implants through different methods. The efforts of many researchers have made great progress in improving the antimicrobial property of implants, but each method has its own inherent drawbacks, which greatly restrict the application.

The coating of implants with materials with excellent antimicrobial properties, such as antimicrobial metal elements, antibiotics, and antimicrobial peptides, has achieved good antimicrobial results, but the safety and long-term effectiveness of the coatings are challenged by the release of metal ions from the implant surface, the rapid consumption and uncontrolled release of drugs, and the resistance to antibiotics. Therefore, more research is needed in the future to develop safer and more effective antimicrobial agents and better controlled release systems to improve the safety and long-term effectiveness of the coating, and the development of material science, manufacturing technology and pharmacology will bring more possibilities for the application of antimicrobial coatings.

Adjusting surface topography for antimicrobial purposes avoids the limitations of chemical modification because it does not consume drugs and relies solely on its surface topography to improve antimicrobial properties, showing promise for long-lasting and safe antimicrobial surfaces, and a variety of techniques have been devised to create surface features and apply them to the design and study of antimicrobial surfaces. However, surface modifications that do not change the surface chemistry at all are practically non-existent, and the contact antimicrobial effect of surface features diminishes with the coverage of conditioned films and the adhesion of bacteria, and it lacks resistance to biofilms that have formed on the surface as well as to uncontacted bacteria. Therefore, dynamic surface features which enable the removal of formed biofilms and stimulus response profiles which enable the reduction of potential toxicity are all worthy directions to be explored.

Implant materials with antimicrobial properties are promising, but are not currently widely used in clinical practice. It is expensive and time-consuming to change the entire implant materials, and many implant materials have shown good antimicrobial properties but have not been well studied in terms of mechanical properties and biocompatibility, so we need to explore these aspects more in the future.

Many materials have only been proven to have antibacterial effects *in vitro*, but not *in vivo*, and few of them have been tested in clinical trials. The peri-implantitis model used in the experiments could not simulate the *in vivo* scenario very well, and most of the experiments only selected specific strains of bacteria, which is a huge difference from the complex microbial environment in the human oral cavity, and the influence of different materials and topographic features on different microorganisms, as well as on the interspecies interaction, need to be studied in more depth. Many experiments have also used only Ti discs as a substitute for implants, which ignores the possible influence of the macroscopic shape of the implant on peri-implant disease and osseointegration. Therefore, the results obtained in the experiments are full of uncertainty as to what results can be obtained in the clinical setting. Before entering clinical trials, we need to build more experimental models that are closer to real-world scenarios to validate these results.

The various methods used to improve the antimicrobial performance of implants require continued efforts by researchers to optimize them. Combining different strategies for synergistic antimicrobial activity is of great attraction, such as the combination of antimicrobial coatings and surface topography that prevent surface adhesion of bacteria and improve the bactericidal effect of antimicrobial agents. The combination of different antimicrobial modalities can reinforce each other to achieve better antimicrobial effects. In addition, while improving the antimicrobial effect of the implant, the improvement of the biocompatibility and mechanical properties of the material should not be neglected. Materials which can improve the antimicrobial performance of the implant, promote the integration of the soft and hard tissues of the host, and match the mechanical properties with the bone tissue are the future directions to be explored.
